# A novel perspective on the evolutionary loss of plasma-accessible carbonic anhydrase at the teleost gill

**DOI:** 10.1242/jeb.246016

**Published:** 2023-10-11

**Authors:** Till S. Harter, Emma A. Smith, Martin Tresguerres

**Affiliations:** Marine Biology Research Division, Scripps Institution of Oceanography, University of California San Diego, La Jolla, CA 92093, USA

**Keywords:** β-NHE, Slc9a1b, Short-circuiting, Haemoglobin, Root effect, Fish

## Abstract

The gills of most teleost fishes lack plasma-accessible carbonic anhydrase (paCA) that could participate in CO_2_ excretion. We tested the prevailing hypothesis that paCA would interfere with red blood cell (RBC) intracellular pH regulation by β-adrenergic sodium-proton exchangers (β-NHE) that protect pH-sensitive haemoglobin–oxygen (Hb–O_2_) binding during an acidosis. In an open system that mimics the gills, β-NHE activity increased Hb–O_2_ saturation during a respiratory acidosis in the presence or absence of paCA, whereas the effect was abolished by NHE inhibition. However, in a closed system that mimics the tissue capillaries, paCA disrupted the protective effects of β-NHE activity on Hb–O_2_ binding. The gills are an open system, where CO_2_ generated by paCA can diffuse out and is not available to acidifying the RBCs. Therefore, branchial paCA in teleosts may not interfere with RBC pH regulation by β-NHEs, and other explanations for the evolutionary loss of the enzyme must be considered.

## Teaser

**Summary:** Extracellular carbonic anhydrase does not interfere with red blood cell beta-adrenergic sodium/proton exchange in an open system, raising new questions about the evolutionary loss of the enzyme in teleost gills.

## INTRODUCTION

Red blood cells (RBCs) are the functional unit of cardiovascular gas transport in vertebrates and contain the principal O_2_-carrying protein, haemoglobin (Hb). In teleost fishes, Hb–O_2_ binding is uniquely pH sensitive, in that proton (H^+^) binding decreases not only Hb–O_2_ affinity by the Bohr effect (Bohr et al., 1904; Jensen et al., 1998), but also Hb–O_2_ carrying capacity by the Root effect (Brittain, 2005; Root, 1931). Teleosts take advantage of this pH sensitivity to greatly enhance O_2_ unloading to their eyes and the swim bladder, by acidifying the blood locally in specialized counter-current exchangers (Pelster, 1997; Wittenberg and Wittenberg, 1962).

However, during a systemic acidosis, which in fish may occur during exercise or hypoxia, teleost Hbs may lose over 50% of their O_2_ carrying capacity (Berenbrink et al., 2011). To prevent severe hypoxemia, the RBCs of many teleosts species can regulate their intracellular pH (pH_i_) against reductions in extracellular pH (pH_e_) (Berenbrink et al., 2005). Under stressful conditions, adrenaline and noradrenaline are released into the blood (Randall and Perry, 1992) and activate beta-adrenergic sodium-proton exchangers (β-NHE) on the RBC membrane that extrude H^+^ to protect pH_i_ and Hb–O_2_ binding (Nikinmaa, 1992). However, the extruded H^+^ can re-equilibrate across the RBC membrane via the Jacobs–Stewart cycle (Jacobs and Stewart, 1942). In the plasma, these H^+^ react with bicarbonate (HCO_3_^−^) to form CO_2_ that diffuses back into the RBC, where it is once again hydrated to produce H^+^, a reaction that is catalyzed by the abundant presence of carbonic anhydrase (CA) within RBCs (Maren, 1967). An adrenergic increase in RBC pH_i_ requires that β-NHE H^+^ extrusion outpaces the re-acidification through the Jacobs–Stewart cycle, which is rate limited by the uncatalyzed formation of CO_2_ in the plasma (Motais et al., 1989a). Therefore, the absence of CA activity in the plasma of teleosts is thought to be essential for RBC pH_i_ regulation and, in fact, a potent plasma CA inhibitor in most vertebrate species seems to ensure that this requirement is met (Henry et al., 1997).

However, plasma accessible carbonic anhydrase (paCA) is present at the tissue capillaries of most vertebrates, where it serves important functions facilitating CO_2_, ammonia and lactate excretion from cellular metabolism into the blood (for review, see Harter and Brauner, 2017). Within the capillaries, a CA4 isoform is anchored to the luminal walls of the endothelium (Damsgaard et al., 2020), which is largely unaffected by endogenous CA inhibitors in the plasma (Gervais and Tufts, 1998; Heming et al., 1993; Roush and Fierke, 1992). Thus, when RBCs reach the capillaries, the formation of CO_2_ in the plasma is catalyzed and the H^+^ influx into the RBCs exceeds the rate of H^+^ extrusion, effectively short-circuiting β-NHE activity (Rummer and Brauner, 2011). The result is a sudden decrease in Hb–O_2_ affinity that enhances the unloading of O_2_ to the tissues. Once the RBCs leave the capillaries and the site of paCA, β-NHE activity recovers pH_i_ during venous transit to safeguard the renewed uptake of O_2_ at the gills (Harter et al., 2018a). This mechanism of β-NHE short-circuiting has been shown to increase the partial pressure of O_2_ (*P*_O_2__) in the swimming muscles of rainbow trout (Rummer et al., 2013) and is critical to sustain maximal exercise performance in Atlantic salmon (Harter et al., 2019). Therefore, interactions between RBC pH_i_ regulation and paCA may be a fundamental aspect of the teleost O_2_ transport system (Harter and Brauner, 2021).

Most vertebrates also have paCA at their gas exchange surfaces (Stabenau and Heming, 2003), where it contributes to CO_2_ excretion by ∼10% in humans (Henry and Swenson, 2000), ∼50% in an elasmobranch (*Squalus acanthias*) (Gilmour et al., 2001) and 100% in an Antarctic icefish (*Champsocephalus gunnari*) that has lost RBCs from the circulation (Harter et al., 2018b). The presence of paCA at the gills of the Pacific hagfish (*Epatretus stoutii*) (Esbaugh et al., 2009) and several elasmobranch species (McMillan et al., 2019) may indicate that this is the ancestral condition. But despite the potential benefits for CO_2_ excretion, most teleost fishes seem to lack paCA at their gills (Harter and Brauner, 2017). It has been proposed that the evolution of highly pH-sensitive Hbs and the need to safeguard branchial O_2_ uptake by RBC β-NHE activity created a selective pressure for the loss of branchial paCA (Randall et al., 2014). However, whether branchial paCA actually interferes with RBC pH_i_ regulation has not been tested experimentally, and fundamental differences between the gill and capillary microenvironments have not been addressed.

At the capillaries, metabolically produced CO_2_ from the tissues diffuses into the blood. This inwardly directed diffusion gradient would prevent any CO_2_ produced by paCA from diffusing out, making it available to acidify the RBCs. Therefore, from the perspective of the RBCs, the tissue capillaries are a functionally closed system with regards to CO_2_ (but not O_2_ that diffuses from the blood to the mitochondria). In contrast, the fish gills are an open system, where most CO_2_ produced by paCA could simply diffuse into the water and would not be available to acidify the RBCs. Therefore, we hypothesized that the presence of paCA in an open system, such as the fish gill, is not an impediment for effective pH_i_ regulation by RBC β-NHE activity.

## MATERIALS AND METHODS

### Animals and husbandry

Rainbow trout [*Oncorhynchus mykiss* (Walbaum 1972)] of both sexes were obtained from Thomas Fish Company (Anderson, CA, USA) and raised in a recirculating freshwater system at 15°C. Juvenile trout (>20 g body mass) were acclimated to seawater by slowly increasing salinity to 35 ppt over the course of 2 weeks. Seawater-acclimated trout were held in 3.5–10 m^3^ tanks in the Hubbs Hall aquatics facility (Scripps Institution of Oceanography), supplied with flow-through seawater at >95% dissolved O_2_. Animals were held for 18 months at a 12 h:12 h light:dark cycle and at a constant water temperature of 12°C, to avoid seasonal effects on RBC β-NHE function (Nikinmaa and Jensen, 1986). Fish were fed twice a week with commercial trout pellets (Skretting Classic Trout, 8 mm sinking) and water quality was monitored daily. Feeding was suspended 48 h before experiments. Fish husbandry and experimental procedures were performed in compliance with the Institutional Animal Care and Use Committee (IACUC) guidelines and were approved by the Animal Care program at the University of California, San Diego (protocol no. S10320).

### Blood sampling

Rainbow trout (705.6±82.0 g) were anaesthetized in seawater containing 70 mg l^−1^ benzocaine (Acros 150785000, Waltham, MA, USA; stock solution made up in ethanol). Once unresponsive, a heparinized syringe was used to draw ∼3 ml of blood from the caudal vein and fish were quickly recovered in their tank for future experiments. Blood was centrifuged (500 ***g*** for 3 min) and the plasma was removed and stored at 4°C overnight. The remaining RBC pellet was rinsed three times with filter sterilized Cortland's saline (in mmol l^−1^: 151 NaCl, 5.1 KCl, 1.6 CaCl, 0.9 MgSO4, 6 NaHCO3, 3 NaH2PO4, 5.6 glucose; at pH 7.8; Wolf, 1963), discarding the buffy coat each time to remove white blood cells and platelets. Finally, the RBCs were resuspended in 25 ml of Cortland's saline and stored with a large headspace on a tilt-shaker overnight at 17°C to reverse the adrenergic effects of sampling stress.

### Open-system experiments

The potential effects of extracellular CA on β-NHE activity at the fish gills were studied using an *in vitro* spectrophotometric approach. RBCs were rinsed in Cortland's saline and suspended in native plasma at 5% haematocrit (Hct), which yields an acceptable optical density for spectrophotometric measurements (Harter et al., 2021). The use of native plasma from rainbow trout was critical, as it: (i) minimizes RBC lysis during tonometry, owing to its higher viscosity compared with saline (Jensen et al., 2009); and (ii) contains a plasma CA inhibitor that effectively neutralises any extracellular CA activity from RBC lysis and is ineffective against CA isoforms from other species, including bovine CA2 (Henry et al., 1997). Blood aliquots of 300 µl were treated with either: (i) a carrier control of 0.25% dimethyl sulfoxide (DMSO, VWR BDH 1115; Radnor); (ii) 10 µmol l^−1^ isoproterenol (ISO; Sigma-Aldrich I6504, St Louis, MO, USA) a β-adrenergic agonist that induces maximal β-NHE activity (Tetens and Lykkeboe, 1988); (iii) ISO plus 10 µmol l^−1^ CA (ISO+CA) from bovine erythrocytes (Sigma C3934≥2500 W-A units), shown to short-circuit RBC β-NHE activity *in vitro* (Rummer and Brauner, 2011); or (iv) ISO plus 1 mmol l^−1^ amiloride (ISO+Am; Sigma A7410) an NHE inhibitor (Mahe et al., 1985). Treated blood aliquots were equilibrated in tonometers (open gas equilibration systems) to a humidified gas mixture (21 kPa *P*_O_2__, 0.3 kPa *P*_CO_2__ at 12°C; gas mixing system GMS, Loligo Systems, Viborg, Denmark) that matches arterial *P*_CO_2__ in normoxic rainbow trout (Brauner et al., 2000). After 1 h, a subsample of 2 µl was taken from the tonometers and Hb–O_2_ binding was studied spectrophotometrically with a Blood Oxygen Binding System (BOBS, Loligo Systems). The remainder of the blood was used to measure blood parameters as described below. All trials were run at 12°C and 21 kPa *P*_O_2__, while *P*_CO_2__ was increased in six steps from 0.3 to 6.0 kPa with 2 min of equilibration in between. During these trials, the absorbance of the blood was recorded once per second at 430 nm, where changes in absorbance correspond to changes in Hb–O_2_ saturation, and at the isosbestic reference wavelength of 390 nm, where absorbance is independent of oxygenation. To calculate Hb–O_2_ saturation from raw absorbance data, a calibration was carried out at the beginning and end of each trial, by equilibrating blood to gas tensions that yielded full Hb oxygenation (99.7 kPa *P*_O_2__, 0.3 kPa *P*_CO_2__) and deoxygenation (0 kPa *P*_O_2__, 0.3 kPa *P*_CO_2__), which was confirmed by examining the absorption spectra.

### Closed-system experiments

The effects of extracellular CA on β-NHE activity in the tissue capillaries were verified *in vitro* using a previously described closed-system preparation (Rummer and Brauner, 2011). Blood sampling and overnight storage were as described above. RBC pellets were rinsed in Cortland's saline and resuspended in native plasma at 25% Hct. The blood was loaded into a tonometer and equilibrated to 7.5 kPa *P*_O_2__ and 0.3 kPa *P*_CO_2__ for 1 h at 12°C. This gas tension yields ∼75% Hb–O_2_ saturation as calculated using previously generated oxygen equilibrium curves. These conditions were chosen so that changes in Hb–O_2_ saturation could be observed in both positive and negative directions during trials (Harter et al., 2018a; Rummer and Brauner, 2011; Shu et al., 2017).

After equilibration, approximately 1 ml of well-mixed blood was loaded into a Micro Respiration Chamber (Unisense, Aarhaus, Denmark), continuously mixed with a stir bar at 350 rpm (Micro Respiration Rack, Unisense) and kept in a temperature-controlled water bath at 12°C. An O_2_ microsensor (OX-200, Unisense) was inserted into the closed vial through a pore and *P*_O_2__ was recorded once per second. After loading, the *P*_O_2__ reading was allowed to stabilize before sequential (every 5 min) injections of: (i) 15 µl of 200 mmol l^−1^ HCl to decrease blood pH by 0.3 units, according to Wood et al. (1982); (ii) 10 µmol l^−1^ ISO to stimulate β-NHE activity; and (iii) 1 µmol l^−1^ CA to short-circuit β-NHE activity. A 10-fold lower concentration of CA was used in the closed system after preliminary trials showed significant effects; thus, in the open system, CA concentration exceeded that required for successful β-NHE short-circuiting. To validate that a respiratory acidosis in the closed system has the same effect as the metabolic acidosis from HCl injections, an additional experiment was run by injecting 20 µl Cortand's saline saturated with CO_2_ (i.e. at 100 kPa or 1,000,000 µatm *P*_CO_2__).

### Blood analysis

Blood parameters were measured after equilibration in tonometers. Hct was measured in triplicate using micro-capillary tubes (Drummond Scientific Company, Broomall, PA, USA) centrifuged at 10,000 ***g*** for 3 min in a Model MB micro-capillary centrifuge (International Equipment Company, Needham Heights, MA, USA). [Hb] was measured on 10 µl of well-mixed blood in 1 ml aliquots of Drabkin's reagent (Sigma D5941). Absorbance of the sample was measured in a UV-VIS spectrophotometer (Shimadsu UV-1820, Columbia, MD, USA) at 540 nm using an absorption coefficient of 10.99 (van Kampen and Zijlstra, 1983). Mean corpuscular Hb concentration (MCHC) was calculated by dividing [Hb] in mmol l^−1^ by Hct (as a fraction) multiplied by 100. Extracellular pH (pH_e_) was measured with a pH microelectrode at 12°C (Fisher Accumet 13-620-850, Hampton, NY, USA; with Denver Instruments UB-10 meter, Bohemia, NY, USA). The remaining blood was centrifuged to separate RBCs and plasma (500 ***g***, 3 min). Plasma total carbon dioxide content (TCO_2_) was measured in triplicate using a Corning 965 (Corning Instruments, Corning, NY, USA), and HCO_3_^−^ was calculated using the Henderson–Hasselbalch equation and published pK_a_ and solubility values for CO_2_ (Boutilier et al., 1984). The RBC pellet was frozen and thawed three times in liquid nitrogen (Zeidler and Kim, 1977) and pH_i_ was measured in the lysate as described above. For RBC fixations, the above tonometry trial was repeated with cells suspended in saline, as plasma proteins can interfere with the quality of antibody staining. RBC fixation and immunostaining were as previously described (Harter et al., 2021), with a custom-made polyclonal antibody raised against rainbow trout β-NHE and a monoclonal mouse anti-Tetrahymena α-tubulin antibody (DSHB12G10).

### Data analysis and statistics

All data were analysed in RStudio v.1.4.1106 using R v.4.0.4 (www.rstudio.org) and the ggplot2 package to generate figures (Wickham, 2009). Raw data from the open-system experiment were generated by dividing the absorbance at 430 nm by the isosbestic absorbance recorded at 390 nm. The last 10 absorbance ratios from each *P*_CO_2__ equilibration step were averaged and used for statistical analysis. Absolute values of Hb–O_2_ saturation were calculated based on the calibration values for fully oxygenated and deoxygenated Hb and applying a linear correction for drift (Harter et al., 2021). The decrease in Hb–O_2_ saturation during the respiratory acidosis was analysed by fitting a nonlinear Hill model to the data (tested against Michaelis–Menten and exponential models and selected based on the lowest Akaike's information criterion; AIC). The two parameters estimated by the Hill model represent the *P*_CO_2__ at which Hb–O_2_ saturation decreases by 50% of the maximal change (EC_50_*P*_CO_2__) and the maximal change in Hb–O_2_ saturation (max. ΔHb–O_2_ sat.). The effects of drug additions on EC_50_*P*_CO_2__ and max. ΔHb–O_2_ sat. were tested with a linear mixed-effects model using individual fish as a random effect (repeated measures). If a significant main effect of treatment (drug additions) was detected, *post hoc* analysis was performed using pairwise *t*-tests with a Benjamini–Hochberg correction of the significance level. The same statistical methods were applied to analyse the blood parameter data (Hct, [Hb], MCHC, pH_e_, pH_i_ and plasma HCO_3_^−^). The raw data from the closed-system experiment were analysed by calculating the change in *P*_O_2__ (Δ*P*_O_2__) after drug treatments, by averaging 10 data points before and after additions of a drug. Whether treatments (HCl, ISO or CA) had an effect on closed-system *P*_O_2__ was tested with paired *t*-tests. Parametric assumptions were confirmed with the Shapiro–Wilk test of normality and Levene's test for homogeneity of variance, and analyses were performed on transformed data if these assumptions were violated. All data are reported as means±s.e.m., for *N=*6 fish (unless stated otherwise).

## RESULTS AND DISCUSSION

### Open-system experiments

**Fig. 1.**
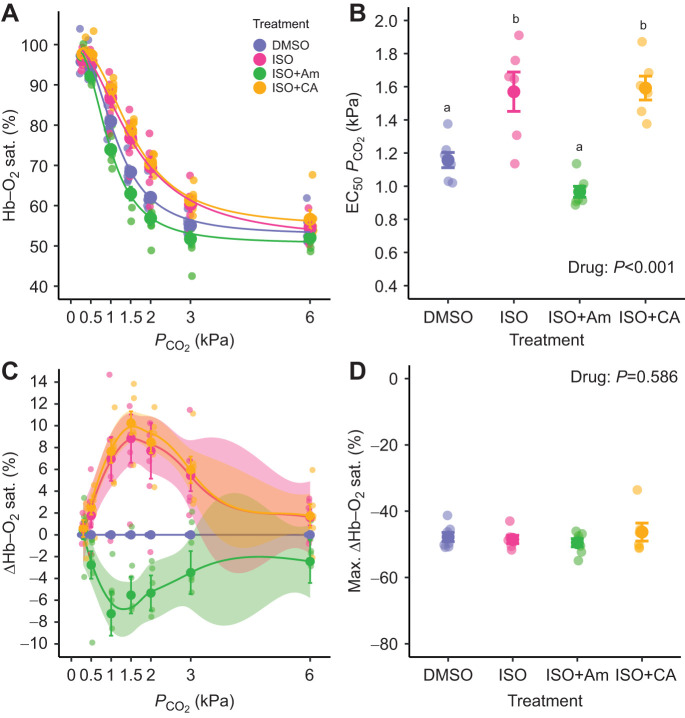
**Results from the open-system experiment.** (A) Changes in haemoglobin–oxygen saturation (Hb–O_2_ sat.; %) of rainbow trout whole blood during a respiratory acidosis (0.3–6 kPa *P*_CO_2__). Blood was treated with either: (i) a carrier control (DMSO; 0.25%), (ii) the β-adrenergic agonist isoproterenol (ISO; 10 µmol l^−1^), (iii) ISO plus amiloride (ISO+Am; 1 mmol l^−1^), an inhibitor of sodium-proton exchangers (NHE), or (iv) ISO plus carbonic anhydrase (ISO+CA; 10 µmol l^−1^). (B) The *P*_CO_2__ at which Hb–O_2_ saturation decreased by 50% of the maximal change (EC_50_*P*_CO_2__; kPa). (C) Changes in Hb–O_2_ saturation relative to paired controls from the same animal (ΔHb–O_2_ sat.; %). (D) The maximal reduction in Hb–O_2_ saturation owing to acidification (max. ΔHb–O_2_ sat.; %). Superscript letters that differ indicate significant differences between treatments. Individual data points, means±s.e.m. and 95% confidence intervals in C (*N*=6–7).

When RBCs were exposed to a respiratory acidosis, Hb–O_2_ saturation decreased significantly in all treatments, as expected owing to an intracellular acidification and the Bohr–Root effect of Hb (Fig. 1A). However, the *P*_CO_2__ at which Hb–O_2_ saturation decreased by 50% of the maximal change (EC_50_*P*_CO_2__; Fig. 1B) was significantly affected by the drug treatments (*P*<0.001). Adrenergically stimulated RBCs in the ISO treatment had significantly higher EC_50_*P*_CO_2__ (1.56±0.13 kPa) compared with DMSO controls (1.14±0.04 kPa). In contrast, stimulated RBCs treated with the NHE inhibitor amiloride (ISO+Am) had a significantly lower EC_50_*P*_CO_2__ (0.96±0.04 kPa) compared with the ISO treatment, and were not different from DMSO controls. Therefore, the increase in Hb–O_2_ saturation after adrenergic stimulation of RBCs can be attributed to β-NHE activity, likely through its well-described effects on RBC pH_i_ (Nikinmaa, 1992); Na^+^ channels that are also affected by amiloride are not involved in the RBC β-NHE response (Reid and Perry, 1994). More importantly, however, the addition of extracellular CA to ISO-treated RBCs (ISO+CA) significantly increased EC_50_*P*_CO_2__ (1.55±0.07 kPa) compared with DMSO and ISO+Am controls and was not different from RBCs treated with ISO alone. This critical finding supports our hypothesis that the presence of extracellular CA in an open system, such as the fish gill, does not impair pH_i_ regulation by RBC β-NHEs. In fact, both ISO- and ISO+CA-treated RBCs maintained Hb–O_2_ saturation ∼10% higher compared with paired DMSO controls from the same animal (Fig. 1C). The negative ΔHb–O_2_ saturation in ISO+AM-treated cells may support the presence of other non-adrenergic NHE isoforms that have been speculated to contribute to RBC pH_i_ protection under routine conditions (Pedersen et al., 2003; Rummer and Brauner, 2011). In all treatments, the protective effects of β-NHE activity on Hb–O_2_ binding decreased at higher *P*_CO_2__ exceeding 3 kPa, where there were no differences in ΔHb–O_2_ saturation between the treatments. This is in line with the finding that the maximal decrease in Hb–O_2_ saturation (max. ΔHb–O_2_ sat.) during the respiratory acidosis was not different between the treatments and was on average −47.8±1.4% (Fig. 1D), which is in line with previous Root effect measurements in rainbow trout (Berenbrink et al., 2011).

**Fig. 2.**
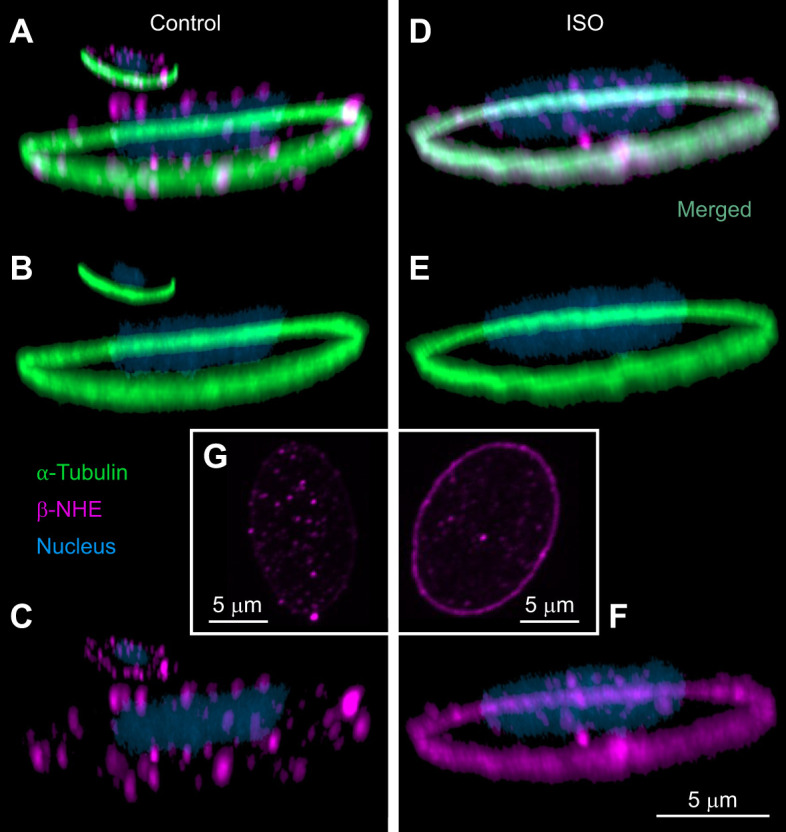
**Immunocytochemical localisation of the β-adrenergic sodium proton exchanger (β-NHE) in rainbow trout red blood cells (RBCs).** Blood was incubated in tonometers with either: (A–C) a carrier control (0.25% DMSO) or (D–F) the β-adrenergic agonist isoproterenol (ISO; 10 µmol l^−1^) for 1 h. Fixed cells were immuno-stained with an anti-α-tubulin antibody to visualize the marginal band (green), with DAPI to visualize the cell nuclei (blue), and with an anti-β-NHE antibody (magenta) raised against the rainbow trout protein. (G) Insets show 2D images highlighting the differences in membrane staining for β-NHE protein between control and ISO-treated RBCs. Representative images for *N*=3 fish.

Our experiments showed robust effects of β-NHE activity on Hb–O_2_ saturation during the spectrophotometric measurements (Fig. 1A–C), dissipating initial concerns about a desensitization of the transporter over the experimental period (Garcia-Romeu et al., 1988). This is in line with previous results where adrenergically stimulated rainbow trout blood was successfully short-circuited by CA additions *in vitro* more than 60 min after it was rinsed to remove ISO (Shu, 2019). Also, RBCs from another teleost, white seabass (*Atractoscion nobilis*), showed β-NHE activity throughout the same protocol used here (Harter et al., 2021). Despite the low Hct of 5%, we observed a trend (*P*<0.10) for RBC swelling in the ISO and ISO+CA treatments, which is indicative of β-NHE activity (Nikinmaa, 1992), and was absent in the ISO+Am or DMSO controls (Fig. S1A). Finally, we found evidence for a translocation of β-NHE protein in adrenergically stimulated rainbow trout RBCs, from the cytoplasm into the membrane (Fig. 2), which is consistent with previous findings in white seabass (Harter et al., 2021) and work describing an increase in radiolabelled β-NHE binding sites in hypoxia-exposed RBCs (Reid and Perry, 1994). The translocation of β-NHE protein in adrenergically stimulated trout RBCs was less prominent than that observed in white seabass RBCs (Harter et al., 2021), perhaps indicating interspecific differences in this novel cellular mechanism that are worthy of investigation. The 3D reconstructions of trout RBC images provide novel insights indicating that β-NHE protein is not homogeneously distributed throughout the RBC membrane, but is most abundant along the marginal band of the cells (Fig. 2). An involvement of cytoskeletal structures, such as α-tubulin, in the intracellular translocation of RBC membrane proteins seems likely, yet remains to be substantiated in dedicated experiments.

*In vitro* studies of β-NHE function do not accurately replicate the conditions of flow, shear and residence time of the RBCs at the gills, factors that warrant some careful consideration. Our findings are generally in line with two *in vivo* studies that injected extracellular CA into rainbow trout exposed to hypoxia (Lessard et al., 1995) or during maximal exercise (Wood and Munger, 1994) and found no changes in arterial O_2_ transport that would be expected owing to β-NHE short-circuiting at the gills. However, the injections of CA *in vivo* also caused significant changes in blood *P*_CO_2__, pH_e_ and the release of catecholamines, which are known to affect β-NHE activity in rainbow trout (Nikinmaa, 1992). Owing to these confounding factors, it can be difficult to isolate the effects of CA on β-NHE function *in vivo*, highlighting the importance of mechanistic *in vitro* experiments. For instance, Motais et al. (1989a,b) found that additions of extracellular CA to adrenergically stimulated rainbow trout blood in tonometers, which are open systems, did not impair the protective effects of β-NHE function on RBC pH_i_; this is in line with our findings. However, very similar experiments by Nikinmaa et al. (1990) showed that the addition of extracellular CA abolished the H^+^ gradient across the RBC membrane, which indicates β-NHE short-circuiting. The discrepancy between these results has not been resolved experimentally, but may be related to the variable diffusive characteristics of tonometry systems.

Upon β-NHE activation in tonometers, H^+^ ions are extruded from the RBC, which increases pH_i_ and decreases pH_e_. In the plasma, H^+^ ions react with HCO_3_^−^ to form CO_2_, which can take one of two routes: some CO_2_ may diffuse out of the open system, driving the dehydration of more HCO_3_^−^, while the consumption of H^+^ leads to a progressive recovery of pH_e_; and some CO_2_ may diffuse into the RBCs, where it is hydrated by intracellular CA to form H^+^ and HCO_3_^−^, which are exported back into the plasma by the β-NHE and anion exchanger, respectively. This recycling of H^+^ through the RBC continuously fuels the β-NHE system and delays the recovery of pH_e_ beyond the time course predicted by the uncatalyzed formation of CO_2_ (Motais et al., 1989a), which has also been reported in rainbow trout *in vivo* (Perry and Thomas, 1993). Because the Jacobs–Stewart cycle is rate-limited by the slow formation of CO_2_ in the plasma, the H^+^ influx into RBCs is slower than H^+^ extrusion by the β-NHE, and pH_i_ remains protected. When extracellular CA is added to a closed system, the Jacobs–Stewart cycle accelerates sufficiently to overwhelm β-NHE H^+^ extrusion, leading to a decrease in RBC pH_i_. However, in an open system, the diffusive loss of CO_2_ diverts H^+^ away from the RBCs, which may prevent changes in RBC pH_i_. Although tonometers are nominally open systems, they are not particularly efficient gas exchangers, as evidenced by transient changes in blood *P*_O_2__ and *P*_CO_2__ upon adrenergic stimulation (Perry and Thomas, 1993). Therefore, the divergent results of Nikinmaa et al. (1990) and Motais et al. (1989a,b) could be explained by different gas transfer efficiencies between the blood and the equilibration gas, where the degree of RBC short-circuiting upon CA addition will depend on how much CO_2_ is lost from the system and how much is available to acidify the cells.

To avoid the diffusive limitations of tonometers, we used a spectrophotometric approach to study β-NHE function through direct measurements of Hb–O_2_ binding. Therefore, 2 µl of blood was spread onto a temperature-controlled glass plate and the large surface-area-to-volume ratio of these samples allowed for an efficient equilibration of gases, as indicated by Hb–O_2_ saturations that responded within seconds after gas tensions were changed. Under these improved experimental conditions, the presence of extracellular CA had no effect on the protective function of β-NHE activity on Hb–O_2_ binding. The situation at the fish gill is likely similar, where RBCs squeeze through thin gill lamellae with large surface areas and most CO_2_ diffuses from the blood to the environment within seconds (Hughes, 1984).

### Closed-system experiments

**Fig. 3.**
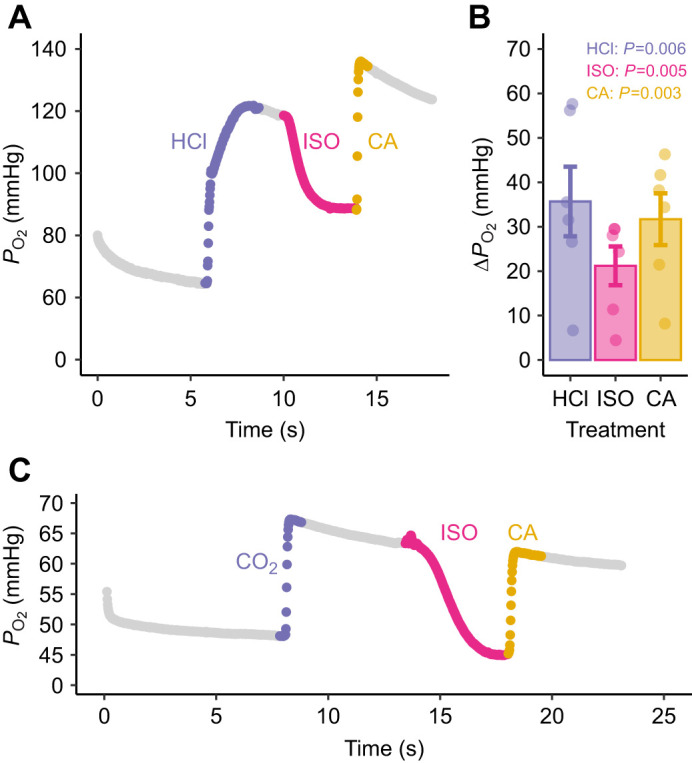
**Results from the closed-system experiment.** (A) Representative trace of the partial pressure of oxygen (*P*_O_2__) in rainbow trout whole blood after sequential injections of: (i) 200 mmol l^−1^ HCl to acidify the blood by 0.3 pH units, (ii) the β-adrenergic agonist isoproterenol (ISO; 10 µmol l^−1^) and (iii) carbonic anhydrase (CA; 1 µmol l^−1^); all had significant effects on *P*_O_2__ (*P*<0.05). (B) Changes in *P*_O_2__ (Δ*P*_O_2__) owing to drug additions, showing individual data points and means±s.e.m. (*N*=6). (C) Representative trace of a closed-system measurement of *P*_O_2__ during a respiratory acidosis induced by injecting 20 μl CO_2_ saturated saline (i.e. 100 kPa *P*_CO_2__), followed by ISO and CA injections as described above.

Our main finding that RBC β-NHE activity can protect pH_i_ in an open system, even in the presence of extracellular CA, does not contradict previous work on RBC short-circuiting in closed systems (Harter et al., 2018a; Rummer and Brauner, 2011; Shu et al., 2017). In fact, we were able to replicate previous findings with our seawater-acclimated rainbow trout and using the same drug aliquots used in the open-system experiments, and even 10-fold lower (1 versus 10 µmol l^−1^) CA concentrations (Fig. 3A). CA additions to adrenergically stimulated RBCs in a closed system caused a significant increase in *P*_O_2__ (Δ*P*_O_2__ 31.7±7.2 mmHg; *P*<0.003; Fig. 3B), indicating that β-NHE activity was unable to protect Hb–O_2_ binding. Furthermore, to validate that β-NHE activity during a respiratory acidosis can also be short-circuited by CA, a separate experiment acidified RBCs by injecting a CO_2_-saturated saline (Fig. 3C). The results were qualitatively similar to the HCl injections, albeit with a smaller Δ*P*_O_2__. This corresponded with the lower acid load from CO_2_ (0.9 µmol l^−1^ H^+^ from CO_2_ versus 3.0 µmol l^−1^ H^+^ from HCl), which is constrained by the solubility of the gas in water. The combined results support that both metabolic and respiratory acidosis in a closed system can create conditions that enable β-NHE short-circuiting.

At the tissue capillaries, metabolically produced CO_2_ diffuses into the blood. Thus, any CO_2_ produced in the plasma cannot diffuse out until blood *P*_CO_2__ becomes so high that it reverses the diffusion gradient from the tissues. Therefore, the tissue capillaries act as a functionally closed system for CO_2_, where paCA can effectively short-circuit β-NHE activity. The resulting decrease in RBC pH_i_ during capillary transit greatly enhances the unloading of O_2_ from pH-sensitive Hb, and may be a fundamental aspect of the O_2_ transport system in many teleost fishes (Harter and Brauner, 2017). The present work adds to this growing body of literature by showing that paCA alone is not sufficient to disrupt RBC pH_i_ regulation and that the diffusive characteristics of the system must also be considered.

### Conclusion and perspectives

The sequence of evolutionary events that led to the complex cardiovascular O_2_ transport system in teleosts has been described in detail (Berenbrink et al., 2005), but the timeline for a loss of branchial paCA remains largely unresolved. Interestingly, however, bowfin (*Amia calva*), a basal actinopterygian without a RBC β-NHE, does not have paCA at the gills (Gervais and Tufts, 1998), perhaps indicating that the loss of the enzyme preceded the evolution of a β-NHE. Therefore, in addition to our mechanistic data, this independent line of evidence supports the idea that the selective pressures that led to the loss of branchial paCA in teleosts did not include the need to protect RBC pH_i_ regulation by β-NHEs. Future work should investigate additional species that bracket the transition from basal actinopterygians to early teleosts, and consider alternative selective pressures for the notorious loss of paCA at their gills.

## Supplementary Material

10.1242/jexbio.246016_sup1 Supplementary information
