# Supplementary material for: A novel perspective on the evolutionary loss of plasma-accessible carbonic anhydrase at the teleost gill
Source: J Exp Biol. 2023 Oct 11;226(19):jeb246016. doi: 10.1242/jeb.246016 (PMC10629482; doi:10.1242/jeb.246016)
Supplement: Supplementary information [file jexbio-226-246016-s1.pdf]

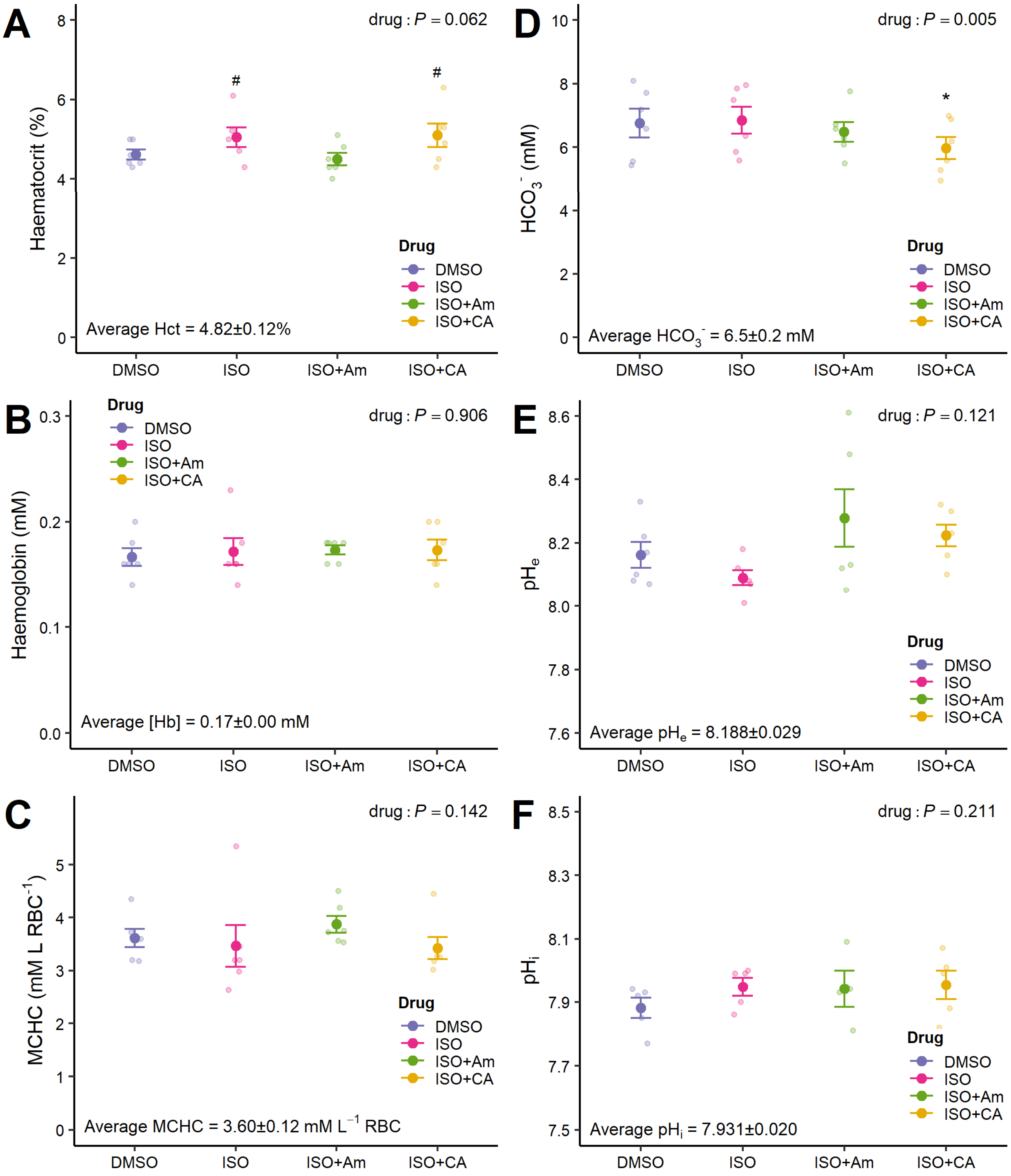

Journal of Experimental Biology • Supplementary information

Fig. S1. Blood parameters of rainbow trout whole blood after equilibration in tonometers. A) Haematocrit (Hct; %), B) haemoglobin concentration ([Hb]; mM), C) mean corpuscular haemoglobin concentration (MCHC; mM haemoglobin L^{-1} red blood cells), D) plasma bicarbonate concentration (HCO_3^-; mM), E) extracellular pH (pH_e) and F) red blood cell intracellular pH (pH_i). Blood was prepared in native plasma at a Hct of 5%, equilibrated in tonometers at 21 kPa PO_2 and 0.3 kPa PCO_2 and treated with either: i) a carrier control (DMSO; 0.25%), ii) the β-adrenergic agonist isoproterenol (ISO; 10 μM), iii) ISO plus amiloride (ISO +Am; 1 mM), an inhibitor of sodium-proton exchangers (NHE) or iv) ISO plus carbonic anhydrase (ISO+CA; 10 μM). The main effect of treatments (drug) was analysed with a linear mixed-effects model ($P < 0.05$). Post-hoc analysis was performed using pairwise t-tests with a Benjamini-Hochberg correction. Significant differences between treatments and DMSO controls are indicated by # ($P < 0.10$) or * ($P < 0.05$). The average values during the trials are shown at the bottom of each panel. All data are means±s.e.m. for $N = 6$ fish (except for pH_i where $N =$ 4-6).

Journal of Experimental Biology • Supplementary information
